# On the role of social position on extreme stress appraisal: Implications for post-traumatic stress disorder

**DOI:** 10.1192/j.eurpsy.2021.1197

**Published:** 2021-08-13

**Authors:** D. Mac Gillavry

**Affiliations:** Leadership, University of Defence, Brno, Czech Republic

**Keywords:** post-traumatic stress disorder, Social context, beta-endorphin, oxytocin

## Abstract

**Introduction:**

Recently, several studies have implicated the social context during a traumatic experience in susceptibility to, and severity of, post-traumatic stress disorder (PTSD). Although the precise mechanisms through which the social context affects the development of PTSD are unknown, it has been suggested that the neuropeptides oxytocin and β-endorphin may play a key role in this dynamic through their effects on both the locus coeruleus and the mesocortical and mesolimbic dopamine systems.

**Objectives:**

This experiment aims to identify in how far a formal social position, endowed by a recognised authority, modulates the stress response in cadets at the Czech military academy during a highly stressful training exercise.

**Methods:**

As part of survival training, 40 cadets partake in a simulation of an avalanche. Although the maximum duration of the experience (being buried under snow) is 15 minutes, most cadets do not last longer than a few minutes with a significant portion requesting termination after a matter of seconds. During the experience, participants are fitted with a heart-rate and heart-rate variability monitor and tested before and after for pain resilience (a common proxy measure for β-endorphin). Participants are randomly allocated to have their individual scores or the average of their collective scores (in small groups of 5) incorporated in their final evaluation of the exercise.

**Results:**

Not all data has been collected yet.

**Conclusions:**

We expect to see a difference in resilience (measured in duration) between the two groups which is mirrored in the afore mentioned biomarkers.

